# Helping someone with problem drinking: Mental health first aid guidelines - a Delphi expert consensus study

**DOI:** 10.1186/1471-244X-9-79

**Published:** 2009-12-07

**Authors:** Anna H Kingston, Anthony F Jorm, Betty A Kitchener, Leanne Hides, Claire M Kelly, Amy J Morgan, Laura M Hart, Dan I Lubman

**Affiliations:** 1 Orygen Youth Health Research Centre, Centre for Youth Mental Health, University of Melbourne, 35 Poplar Rd (Locked Bag 10), Parkville, Victoria 3052, Australia

## Abstract

**Background:**

Alcohol is a leading risk factor for avoidable disease burden. Research suggests that a drinker's social network can play an integral role in addressing hazardous (i.e., high-risk) or problem drinking. Often however, social networks do not have adequate mental health literacy (i.e., knowledge about mental health problems, like problem drinking, or how to treat them). This is a concern as the response that a drinker receives from their social network can have a substantial impact on their willingness to seek help. This paper describes the development of mental health first aid guidelines that inform community members on how to help someone who may have, or may be developing, a drinking problem (i.e., alcohol abuse or dependence).

**Methods:**

A systematic review of the research and lay literature was conducted to develop a 285-item survey containing strategies on how to help someone who may have, or may be developing, a drinking problem. Two panels of experts (consumers/carers and clinicians) individually rated survey items, using a Delphi process. Surveys were completed online or via postal mail. Participants were 99 consumers, carers and clinicians with experience or expertise in problem drinking from Australia, Canada, Ireland, New Zealand, the United Kingdom, and the United States. Items that reached consensus on importance were retained and written into guidelines.

**Results:**

The overall response rate across all three rounds was 68.7% (67.6% consumers/carers, 69.2% clinicians), with 184 first aid strategies rated as essential or important by ≥80% of panel members. The endorsed guidelines provide guidance on how to: recognize problem drinking; approach someone if there is concern about their drinking; support the person to change their drinking; respond if they are unwilling to change their drinking; facilitate professional help seeking and respond if professional help is refused; and manage an alcohol-related medical emergency.

**Conclusion:**

The guidelines provide a consensus-based resource for community members seeking to help someone with a drinking problem. Improving community awareness and understanding of how to identify and support someone with a drinking problem may lead to earlier recognition of problem drinking and greater facilitation of professional help seeking.

## Background

The global consumption of alcohol is growing at a rapid rate, making it the fifth leading risk factor for avoidable disease burden [1]. The health and social costs of problem drinking (i.e., alcohol abuse or dependence) impact both the drinker and society at large [2], highlighting the need for a broad community-based response that includes both government-led primary prevention and community-level interventions.

Research suggests that community interventions can play an integral role in addressing problem drinking. For instance, there is growing evidence that the social networks of individuals with a drinking problem are an important source of support and assistance [3]. Often however, social networks do not have adequate knowledge about mental disorders (including substance use) or how to treat them (i.e., poor mental health literacy), affecting their ability to respond effectively. Although there is a broad range of information about problem drinking available to the public (e.g., internet and printed resources), the content is often inconsistent, or even inaccurate, with little evidence for its effectiveness [3]. The community's lack of mental health literacy is concerning as the response that a drinker receives from their social network can have a substantial impact on their willingness to seek help [4]. Being aware of when and how to encourage a drinker to seek appropriate help is an important community skill, especially as the majority of problem drinkers do not seek help [5]. Not seeking help increases the harms associated with problem drinking, such as developing co-morbid physical and mental health problems [6].

In response to poor mental health literacy within the community, Kitchener and Jorm [7] developed a Mental Health First Aid (MHFA) training program. MHFA is defined as the help provided to a person who may have, or may be developing, a mental health problem (such as problem drinking), or is in a mental health crisis. Similar to first aid, which is designed to educate the public about an appropriate first response to someone with a physical disorder or injury, MHFA educates people about an appropriate response to someone with a mental health problem or in a crisis [8]. Within the MHFA training program, first aid for problem drinking is defined as the help provided to someone who may be developing, or may already have, a drinking problem, or is in an alcohol-related crisis (e.g., alcohol poisoning). MHFA is given until appropriate professional help is received or until the crisis resolves.

A suite of MHFA guidelines has been developed using expert consensus to identify strategies for mental health problems and crises addressed within the MHFA training program [9-14]. Guidelines already developed are: first aid for depression, psychosis, panic attacks, suicidal thoughts and behaviours, non-suicidal self-injury, child and adult trauma and eating disorders (see http://www.mhfa.com.au/Guidelines.shtml). Expert consensus (viz. the Delphi process) was used to identify suitable first aid strategies, as randomized controlled trials of component first aid strategies are not feasible. The Delphi process involves a group of experts making private/independent ratings on a series of items. The experts receive a statistical summary showing how the entire group rated the items and are asked to reconsider their original ratings (which are also provided) in light of this feedback - the experts can either maintain or change their original items [15].

To identify first aid strategies for problem drinking that are suitable for members of the general public to carry out, the present study sought consensus across clinicians, consumers and carers with expertise in, or experience with, problem drinking. Consumer and carer perspectives were included as their lived experience involves many aspects of a first aider's role, and they therefore represent people who might typically receive or give first aid. It is thought that agreement among these different perspectives provides best practice for MHFA.

## Methods

### Literature search

A systematic literature review was conducted by one of the authors (A.H.K) of websites, books and journal articles for strategies about how to help someone who may be developing, or may have, a drinking problem. This involved a comprehensive internet search using Google search engines (http://www.google.com, http://www.google.co.uk and http://www.google.com.au). The following search terms were entered into each: *alcohol *or *alcoholic *and *intoxication*, *alcohol poisoning*, *binge drinking*, *alcohol abuse*, *alcohol dependence*. The first 50 sites for each set of search terms were examined for strategies about how to help someone with a drinking problem. This technique yielded 250 sites per search engine. Any links that appeared on these web pages that were thought may contain useful information were followed. Relevant journal articles published between January 1997 and December 2007 were sought from PsycINFO and PubMed. This yielded 997 and 1572 articles respectively, which were then scanned for any relevance to first aid. The 50 most popular books on the Amazon website published from 1980 onwards were also selected and reviewed. Following this extensive review of the literature, suggestions for first aid actions were obtained from approximately 45 websites, 3 books and 7 journal articles. The majority of first aid actions came from websites, as few books and journal articles focused on pre-clinical interventions.

### Survey development

The information gathered from these sources was analysed by one of the authors (A.H.K) and written into first aider action statements that could be presented to the panels for rating. These statements were first presented to a working group, who screened the items to ensure they fitted the definition of MHFA for problem drinking, were comprehensible and had a consistent format (with the aim of remaining as faithful as possible to the original meaning and wording of the information). After several draft surveys, the group identified 285 items that formed the Round 1 survey. The Round 1 survey was organized around five main sections. (1) The *problem drinking *section included items about recognizing and understanding problem drinking, approaching the person, managing the person's unwillingness to change, and facilitating and managing resistance to seeking professional help. (2) The *low-risk drinking *section included items about understanding low-risk drinking, encouraging the person to drink at lower levels, providing practical tips on doing so, encouraging other supports, and dealing with social pressure to drink. (3) The *alcohol intoxication *section included items about recognizing and understanding alcohol intoxication, helping an intoxicated person, talking to them, getting them home, and managing aggression. (4) The *emergencies related to alcohol intoxication *section included items about general principles of assisting in an emergency, seeking medical help, and managing vomiting, drowsiness, alcohol poisoning and other alcohol-related emergencies. (5) The *alcohol withdrawal *section included items about severe alcohol withdrawal. Comment boxes were included in the Round 1 survey, which allowed panel members to comment and give feedback after each section.

### Panel formation

Consumers, carers and clinicians with expertise or experience in problem drinking were recruited from Australia, Canada, Ireland, New Zealand, the United Kingdom, and the United States. Clinical experts (panel one) approached were international authorities on problem drinking, as well as experienced senior clinicians working within alcohol and other drug treatment settings. Clinical experts were recruited through direct email contact with members of the international editorial boards of the top seven peer-reviewed substance use journals, addiction specialist colleges and societies, and major addiction treatment centres in each country. Consumers (people with a past history of problem drinking) and carers (people with experience caring for someone with problem drinking) were integrated into a second panel as there were not sufficient numbers to divide them into separate panels (Delphi convention recommends a minimum of 15 members per panel [16]). Consumers and carers were recruited by distributing information about the study to consumer and carer organizations associated with alcohol and drug and/or mental health problems in each country. Consumers and carers with experience in an advocacy role were targeted, to ensure that participants had an understanding of problem drinking beyond their own personal experience. Consumers and carers who had authored books about their experience with problem drinking were also invited to participate. No attempt was made to make panels representative. The Delphi method does not require representative sampling; it requires panel members who are information- and experience-rich.

Ninety-nine panel members were recruited from Australia (14 consumers/carers, 39 clinicians), Canada (6 consumers/carers, 6 clinicians), Ireland (1 clinician), New Zealand (1 consumer, 2 clinicians), the United Kingdom (8 consumers/carers, 9 clinicians) and the United States (5 consumers/carers, 8 clinicians). Fifty-three participants were female (68% of the consumers and carers, 46% of the clinicians). The age of consumers and carers ranged from 18-60+ years (median age category was 50-59 years), while the age of clinicians ranged from 30-60+ years (median age category was 40-49 years).

Once participants agreed to participate in the study, they were given the option of completing the surveys online (using SurveyMonkey, http://www.surveymonkey.com) or via postal mail. The study was approved by the Human Research Ethics Committee at the University of Melbourne.

### The Delphi process

The Delphi process was used to survey expert opinion. This was achieved by asking panel members to rate the importance of potential first aid strategies, bearing in mind that a first aider was a member of the general public and therefore did not necessarily have a medical or clinical background. The rating scale used was *essential*, *important*, *don't know/depends*, *unimportant *and *should not be included*. *Not qualified to answer *was included in the rating scale in section 4 of the survey. On completion of each round (there was a total of three rounds), the survey responses were analysed by obtaining percentages for the consumer/carer and clinician panels for each item. The following cut-off points were used:

#### Criteria for accepting an item

• If at least 80% of both the consumer/carer and clinician panels rated an item as essential or important as a MHFA guideline for problem drinking, it was included in the final guidelines.

#### Criteria for re-rating an item

• If 80% or more of the panel members in only one group rated an item as essential or important as a MHFA guideline for problem drinking, we asked all panel members to re-rate that item in the next round.

• If 70%-79% of panel members from both groups rated an item as essential or important, we asked all panel members to re-rate that item.

• Items were re-rated once only. If an item was not endorsed after two rounds it was excluded from the guidelines.

#### Criteria for rejecting an item

• Any items that did not meet the above three conditions were excluded.

After each Round, each panel member was sent a report describing how the results had been analysed and listing all items endorsed in that Round as MHFA guidelines. The report also contained items that required re-rating, accompanied by a summary (as a percentage) of each panel's ratings and the panel member's previous rating for each item. In light of this feedback, panel members were asked to maintain or modify their original ratings in the next Round. In addition, the Round 1 report also contained new items generated through panel members' comments to be rated for the first time.

To analyse the comments that panel members had written during the Round 1 survey, one of the authors (A.H.K) reviewed the comments and wrote them up as first aid strategies. The working group evaluated the suggested strategies to determine whether they were original ideas that had not been included in the Round 1 survey, and whether they met the criteria for a MHFA item. Any strategy that was judged by the group to be an original idea was included as a new item to be rated in the Round 2 survey.

## Results

**Figure 1:**
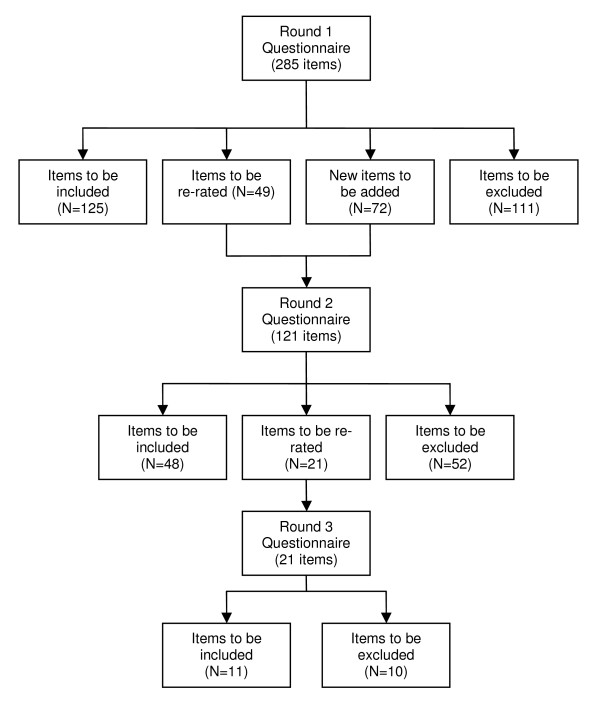
**Overview of items included, excluded, created and re-rated in each round of the survey**.

The overall response rate (those who participated in all three rounds) was 68.7% (67.6% consumers/carers, 69.2% clinicians). See Table 1 for the number of panel members who completed each round. Figure 1 shows an overview of the numbers of items that were included, excluded, created and re-rated in each round of the survey.

**Table 1:** Participant numbers for each round of the survey

	*Round 1* n	*Round 2* n (%)	*Round 3* n (%)
Consumer/Carers	34	27 (79%)	23 (68%)
Clinicians	65	50 (77%)	45 (69%)

Total	99	77 (78%)	68 (69%)

Across the three rounds, 184 first aid strategies were rated as *essential *or *important *by ≥80% of the panel members in each of the two groups (see Additional File 1). One of the authors (A.H.K) prepared a draft of the final guidelines document by grouping items of similar content under specific headings. The items were strung together into prose so that the guidelines offered the first aider a coherent approach to MHFA for problem drinking. The working group improved this draft before it was given to panel members for final comment, feedback and endorsement. Any comments made by panel members were presented to the working group and integrated into the document if deemed relevant and appropriate. See Additional File 2 for the MHFA guidelines for problem drinking.

## Discussion

This study is part of a larger research program using the Delphi process to develop a suite of Mental Health First Aid (MHFA) guidelines designed to inform community members on how to help someone who may have, or may be developing, a mental health problem or is in a mental health crisis [9-14] .

The MHFA guidelines for problem drinking are the only known resource to have identified strategies for helping someone with a drinking problem based on consensus between clinicians, consumers and carers. Despite the unique perspective each panel brought to the guidelines, consensus was reached on a large proportion of strategies. The panels reached consensus on strategies to: recognize problem drinking; approach someone if there is concern about their drinking; support that person to change their drinking and how to respond if they are unwilling to change; facilitate professional help seeking and respond if professional help is refused; and manage an alcohol-related medical emergency.

The panels agreed that the guidelines should include strategies for assisting people drinking at high-risk levels, as well as individuals meeting criteria for alcohol abuse or dependence (as defined by DSM-IV [17]). Thus, the guidelines address drinking behaviours (e.g., binge drinking) that are often considered acceptable within many age groups or cultures and may not be identified as problematic. By broadening the community's understanding of problems associated with drinking, it is anticipated that such problems will be identified earlier and professional help sought sooner. The guidelines also address what to do if the person does not respond to the first aider's intervention, including strategies about what to do if the person is unwilling to change their drinking behaviour or access professional help. Three strategies were endorsed regarding behaviours that the first aider should not engage in, such as *the first aider should not cover up the person's drinking or behaviour*. Such strategies encourage the first aider to create an environment that supports the drinker to change their drinking behaviour. Creating an environment that helps the drinker recognize change may be beneficial and may also help them recognize the need for professional help. This may subsequently reduce the delay between the identification of problem drinking and engagement with professional help.

Consistent with other MHFA guidelines (e.g., Suicidal thoughts and behaviours: first aid guidelines; [9]), the problem drinking guidelines endorsed the drinker's autonomy to decide whether professional help is sought and that the first aider's role is only to support the drinker's decision. This is reflected, for example, in the endorsement of the item *The first aider should tell the person that they will *support *them in getting professional help*, and rejection of the item *The first aider should *encourage *the person to seek professional help*. As one clinician commented "*It should not be an aim of the first aider to steer a person in the direction of professional help...The emphasis should be on a discussion of help available should the person begin to indicate that they are receptive to help*". Based on feedback given by some participants, it appears that a universal guideline encouraging the first aider to advocate for professional help was not endorsed for a number of reasons. These included the notions that professional help is not appropriate for all drinkers; the drinker's individual circumstances should be taken into account; the drinker has a right to choose; the drinker's readiness to change should be taken into consideration (it is more important that the first aider ensures the drinker feels they can ask for professional help when they are ready, rather than forcing the issue of professional help when they aren't ready); and the first aider's involvement in facilitating professional help should depend on how much the drinker wants the first aider to be involved.

A strength of the study was the inclusion of comment boxes which allowed panel members to give qualitative feedback about items within the survey. Panel members' explanations about why they rejected an item in Round 1 gave the authors valuable insight, often resulting in the resubmission of an item in Round 2 with different phrasing or emphasis to ensure an important concept was not rejected because of the way it was written. In the section about encouraging low-risk drinking strategies, participants raised concerns (the only time in the survey) about distinguishing between appropriate help for someone who is a high-risk drinker rather than someone who has alcohol abuse/dependence. In particular, there was concern that low-risk drinking strategies are not suitable for someone who is alcohol dependent. For example, a carer wrote, "*It is important to...distinguish between someone who is an 'episodic heavy drinker' or 'alcohol dependent'. Many of these [low-risk drinking] questions are suitable advice for someone who is not dependent on alcohol*". This concern was addressed by a sequence of items in the second round of the survey, encouraging the first aider to only provide information about low-risk drinking to the person if they wanted it. For example, *The first aider should ask the person if they would like some tips on low-risk drinking*. In addition, all low-risk drinking items from Round 1 that required re-rating were submitted to the panel in two forms in Round 2. Items were presented firstly in their original form (e.g., *The first aider should advise the person what a standard drink is*) and secondly prefaced with *If the person wants some advice on low-risk drinking*, (e.g. *If the person wants some advice on low-risk drinking, the first aider should advise the person what a standard drink is*). Thus, rather than presenting the low-risk drinking tips as information that should be given to all drinkers, it was instead presented as information available to the first aider who could use it as deemed appropriate. This approach resulted in many low-risk drinking strategies being endorsed in the second and third rounds.

The guidelines were based on consensus between international panels of clinical experts and consumers/carers. However, the small size of the panels and the difficulty recruiting carers must be acknowledged as limitations of the study. Despite approaching hundreds of organizations across six countries, we were unable to recruit enough carers to have a separate panel of carers. We had set a minimum panel size as 20, consistent with previous Delphi studies [11,12]. One reason for the difficulty in recruitment is that we sought out consumers and carers who were information- and experience-rich, and required that they be in an advocacy role or the author of a relevant book. As a result, we chose to integrate the carers and consumers into one panel despite our awareness that carers and consumers approach problem drinking and first aid from different perspectives. Nevertheless, this study is unusual in recognizing the importance of consumer and carer perspectives and giving them equal weight with clinicians when developing guidelines [18].

Another limitation of the current study is that the guidelines have been developed specifically for Western, English-speaking countries. They therefore may not be applicable to non-Western cultures or to cultural minorities within English-speaking countries. However, there is scope to use the Delphi process to adapt the guidelines to specific cultures [19,20]. This process is currently underway with the MHFA guidelines for problem drinking being adapted for Australian Aboriginal and Torres Strait Islander people.

Finally, although the guidelines are based on consensus of clinical experts, consumers and carers, the effectiveness of the endorsed first aid strategies remains to be tested. The guidelines document needs to be evaluated for its usefulness as a stand-alone source of information, as well as its utility in guiding the content of training programs. To evaluate the guidelines as a stand-alone document, we are currently doing research on whether people who download it from a website http://www.mhfa.com.au get useful information that guides their first aid actions. The guidelines are also being used to develop an improved second edition MHFA training course. Previous trials have shown the effectiveness of MHFA training in improving knowledge, reducing stigmatizing attitudes and increasing helping behaviour [21-26], and no evidence has been found of harms [27]. However, this research was based on the first edition of the MHFA training course that was not based on consensus guidelines. Further studies of MHFA training are warranted to ensure that the actions of first aiders are both practical and helpful, and that there are not unintended harms such as labelling people in a way that might increase stigma and marginalization.

## Conclusion

In conclusion, these guidelines provide first aid strategies that have been agreed upon by an international panel of clinicians, carers and consumers with expertise and/or experience in problem drinking. The guidelines will provide an important resource for community members seeking to help someone with a drinking problem, hopefully leading to earlier recognition and greater facilitation of professional help seeking. Future research should assess the effectiveness of the first aid strategies endorsed within these guidelines to ensure that they increase helping behaviour and reduce stigma.

## Competing interests

The authors declare that they have no competing interests.

## Authors' contributions

AJ and DL designed the study and wrote the protocol with input from BK, CK, LH and AK. AK completed the literature review, initial survey construction, recruitment of participants, data collection and analysis, and prepared drafts of the guidelines. A working group, consisting of AJ, BK, DL, AK, LH, CK, AM and LH, gathered regularly to give feedback and make improvements on each draft of the Rounds 1, 2 and 3 surveys and the final guidelines. AK wrote the first draft of the manuscript with input from DL and AJ. All authors contributed to and have approved the final manuscript.

## Pre-publication history

The pre-publication history for this paper can be accessed here:

http://www.biomedcentral.com/1471-244X/9/79/prepub

## Supplementary Material

Additional file 1 Items that received ≥80% consensus across both the consumer/carer and clinician panels. Click here for file

Additional file 2 **Helping someone with problem drinking: Mental Health First Aid guidelines**. This file may be distributed freely, with the authorship and copyright details intact. Please do not alter the text or remove the authorship and copyright details. Click here for file
