# Supplementary material for: Helping someone with problem drinking: Mental health first aid guidelines - a Delphi expert consensus study
Source: BMC Psychiatry. 2009 Dec 7;9:79. doi: 10.1186/1471-244X-9-79 (PMC2799400; doi:10.1186/1471-244X-9-79)
Supplement: Additional file 1 — Items that received ≥80% consensus across both the consumer/carer and clinician panels. [file 1471-244X-9-79-S1.doc]

Items that received ≥80% consensus across both the consumer/carer and clinician panels

| **First aid strategies for problem drinking** | **Round accepted** |
| --- | --- |
| **Recognising problem drinking** |  |
| The first aider should be able to recognise problem drinking. | 1 |
| The first aider should be able to recognise the signs of high-risk drinking. | 1 |
| The first aider should be able to recognise the symptoms of alcohol abuse. | 1 |
| The first aider should be able to recognise the symptoms of alcohol dependence. | 1 |
| **Understanding problem drinking** |  |
| The first aider should have general knowledge of some of the reasons why people drink alcohol to excess. | 1 |
| The first aider should be aware that the person might not recognise that they have a drinking problem. | 1 |
| The first aider should be aware that the person may deny they have a drinking problem. | 1 |
| The first aider should be aware that forcing the person to admit they have a drinking problem may cause conflict. | 1 |
| The first aider should be aware that the person is the only one who can make the decision to change their drinking behaviour. | 1 |
| The first aider should be aware that a person’s willpower and self-resolve is not always enough to help them stop problem drinking. | 1 |
| The first aider should be aware that it is not easy to change drinking habits. | 1 |
| The first aider should be aware that giving advice alone may not help the person change their drinking behaviour. | 1 |
| The first aider should be aware that detoxification is only part of the recovery process and that many lifestyle changes are required to change drinking behaviours. | 1 |
| The first aider should be aware that the person may relapse once or several times before changing their drinking patterns. | 1 |
| The first aider should be aware that it is possible for the person to change their drinking habits. | 2 |
| The first aider should be aware that it is possible for them to assist the person change their problem drinking. | 2 |
| The first aider should be aware that the person may be drinking as a means of coping with problems in their life (e.g. underlying emotional distress or mental illness). | 2 |
| The first aider should be aware that problem drinking may be related to an untreated mental illness. | 2 |
| The first aider should be aware that mental health problems can be caused or exacerbated by drinking alcohol. | 2 |
| The first aider should be aware that to stop problem drinking, the person’s underlying emotional distress or mental health problems will usually need to be addressed. | 3 |
| **Talking to the person about their drinking** |  |
| The first aider should talk with the person in a quiet, private environment. | 1 |
| The first aider should talk to the person at a time when there will be no interruptions. | 1 |
| The first aider should talk to the person when both are sober. | 2 |
| The first aider should be aware that the person may not recall events that occurred whilst they were intoxicated (i.e. they may have blacked out). | 1 |
| The first aider should talk to the person when both are in a calm frame of mind. | 1 |
| The first aider should interact with the person in a supportive way. | 1 |
| The first aider should try to listen to the person non-judgmentally. | 1 |
| The first aider should not talk in a confrontational way. | 2 |
| The first aider should not use a threatening approach. | 1 |
| The first aider should not lecture the person. | 1 |
| The first aider should avoid expressing moral judgments about the person’s drinking. | 1 |
| The first aider should not label the person (e.g., an 'alcoholic' or 'addict'). | 2 |
| The first aider should use ‘I’ statements, for example, "I am concerned about how much you’ve been drinking lately". | 2 |
| The first aider should identify and discuss the person’s behaviour rather than criticise their character, for example, “Your drinking seems to be getting in the way of your friendships” rather than “You're a pathetic drunk”. | 1 |
| The first aider should not accuse the person of being an alcoholic. | 1 |
| The first aider should consider the person’s readiness to talk about their drinking problem by asking about areas of their life that it may be affecting, for example, their mood, work performance and relationships. | 1 |
| The first aider should talk to the person about their drinking openly and honestly. | 1 |
| The first aider should try to understand the person’s own perception of their drinking. | 1 |
| The first aider should advise the person that alcohol may interact with other drugs (illicit or prescribed) in an unpredictable way which may lead to a medical emergency. | 2 |
| The first aider should encourage the person to find some information on how to reduce the harms associated with their problem drinking. | 2 |
| The first aider should tell the person what they are willing and able to do to help. This may range from simply being a good listener to organising professional help. | 1 |
| The first aider should not expect a change in the person’s thinking or behaviour right away; this conversation might be the first time the person has thought about their drinking as a problem. | 1 |
| The first aider should ask the person about their drinking behaviour, e.g. about how much alcohol the person tends to drink. | 3 |
| The first aider should ask the person if they believe their drinking is a problem. | 3 |
| **What to do if the person is unwilling to change their drinking behaviour** |  |
| The first aider should be aware that they can speak with a health professional to determine how best to approach the person about their concerns. | 2 |
| The first aider should be aware that they can consult with others who have dealt with problem drinking about effective ways to help the person. | 2 |
| The first aider should discuss with the person the link between their drinking behaviour and the negative consequences. | 2 |
| If the person is unwilling to change their drinking behaviour, the first aider should |  |
| ... not join in drinking with them. | 1 |
| ... not try to control them by bribing, nagging, threatening or crying. | 1 |
| ... not feel guilty or responsible. | 1 |
| … not cover up or make excuses for them. | 2 |
| … take on the person's responsibilities except if not doing so would cause harm (e.g. to their own or other’s lives). | 3 |
| **Professional help** |  |
| The first aider should be aware that the person cannot be forced to get professional help except under certain circumstances, for example, if a violent incident results in the police being called or following a medical emergency. | 1 |
| The first aider should know the following warning signs indicate the person needs professional help… |  |
| The person acknowledges they think a lot about alcohol and when they’ll next get a chance to drink. | 1 |
| The person is in debt because of the amount of money they spend on alcohol. | 1 |
| The person becomes anxious when they cannot get access to alcohol. | 1 |
| The person needs alcohol to help deal with certain situations. | 1 |
| The person gets into arguments or has accidents because of alcohol. | 1 |
| The person’s ability to perform day-to-day tasks is severely disrupted. | 1 |
| **Discussing professional help with the person** |  |
| The first aider should be aware that the person may not want professional help when it is first suggested to them. | 1 |
| The first aider should be aware that the person may find it difficult to accept professional help. | 1 |
| The first aider should be aware that it is ultimately the person’s decision to get professional help. | 1 |
| The first aider should explain to the person that there are several approaches available for treating drinking problems. | 1 |
| The first aider should reassure the person that professional help is confidential. | 1 |
| The first aider should tell the person that they will support them in getting professional help. | 1 |
| If the person is willing to seek professional help, the first aider should give them information about local options. | 1 |
| If the person is willing to seek professional help, the first aider should encourage the person to make an appointment. | 1 |
| **What to do if the person unwilling to get professional help** |  |
| If the person is unwilling to get professional help, the first aider should |  |
| ... be compassionate and patient while waiting for the person to accept they need it. | 1 |
| ... be prepared to talk to them again in the future. | 1 |
| … set boundaries around what behaviour they are willing and not willing to accept from the person. | 2 |
| If the person is unwilling to get professional help, the first aider because they don’t want to stop drinking completely, the first aider should explain that the treatment goal may be to reduce alcohol consumption rather than to quit altogether. | 2 |
| The first aider should continue to suggest professional help to the person if they are putting themselves or others at risk of harm. | 2 |
| **Understanding low-risk drinking** |  |
| The first aider should be aware that abstinence from drinking may not be the person’s goal and that reducing the quantity of alcohol consumed is a worthwhile objective. | 1 |
| The first aider should be familiar with national guidelines for low-risk alcohol consumption. | 1 |
| The first aider should know what a standard drink is. | 1 |
| **How to encourage low-risk drinking** |  |
| The first aider should tell the person that only they can take responsibility for reducing their alcohol intake. | 1 |
| The first aider should tell the person that changing drinking patterns is difficult but they should not give up trying. | 1 |
| **Practical tips for low-risk drinking** |  |
| The first aider should ask the person if they would like some tips on low-risk drinking. | 3 |
| If the person wants to change their drinking behaviour, the first aider should suggest tips for low-risk drinking. | 2 |
| If the person wants some advice on low-risk drinking, the first aider should … |  |
| … tell the person where they can get information about low-risk drinking. | 2 |
| … tell the person where they can access it. | 2 |
| …advise the person what a standard drink is. | 2 |
| … should inform the person that the number of standard drinks is often listed on the beverage's packaging. | 3 |
| … advise the person to be aware of the number of standard drinks they consume. | 2 |
| … advise the person not to let people top up their drink before it is finished, so they don’t lose track of how much alcohol they have consumed. | 2 |
| … advise the person to eat while drinking. | 2 |
| … advise the person to drink plenty of water on a drinking occasion to prevent dehydration. | 3 |
| … advise the person to reduce the amount of alcohol they drink by consuming drinks with lower alcohol content (for example, drinking light beer instead of full strength beer). | 3 |
| … advise the person to switch to non-alcoholic drinks when they start to feel the effects of alcohol. | 2 |
| … advise the person to avoid drinking competitions and drinking games. | 2 |
| … advise the person to drink slowly, for example, by taking sips instead of gulps and putting their drink down between sips. | 2 |
| … advise the person to have one drink at a time. | 2 |
| … advise the person to think of drinking alcohol as complementary to another activity instead of the sole activity. | 3 |
| … advise the person to spend their time in activities that don’t involve drinking. | 2 |
| The first aider should advise the person to avoid keeping up with their friends drink for drink. | 1 |
| The first aider should advise the person to be aware of the alcohol content of their drink. | 1 |
| The first aider should advise the person to identify situations where they are most likely to drink and to avoid them if practical. | 1 |
| **Encouraging other supports** |  |
| The first aider should make the person aware of the range of non-professional supports available for problem drinking, for example, self-help groups. | 2 |
| The first aider should encourage the person to reach out to friends and family who support their efforts to change their drinking behaviours. | 1 |
| The first aider should encourage the person to spend time with non-drinking family and friends who support the person's effort to change their drinking behaviour. | 2 |
| The first aider should encourage the person to spend time with supportive non-drinking friends and family. | 3 |
| The first aider should warn the person that not all family and friends will be supportive of their efforts to change their drinking behaviour. | 1 |
| **Dealing with social pressure to drink** |  |
| The first aider should be aware that there is often social pressure to get drunk when drinking. | 1 |
| The first aider should advise the person to be assertive when they feel pressured to drink more than they want or intend to. | 1 |
| The first aider should tell the person that they have the right to refuse alcohol when under pressure to drink. | 1 |
| The first aider should tell the person that they can say “no thanks”, without explanation, when under pressure to drink. | 1 |
| The first aider should suggest different ways the person can say no when pressured to drink such as “I don’t feel like it”, "I don't feel well" or "I am taking medication”. | 2 |
| The first aider should encourage the person to practice different ways of saying ‘no’ to social pressure to drink. | 2 |
| The first aider should suggest to the person that the people who care about them will accept their decision not to drink. | 2 |
| **Recognising alcohol intoxication** |  |
| The first aider should be able to recognise the signs of alcohol intoxication. | 1 |
| The first aider should be aware of the major factors that account for variation in symptoms of intoxication, such as prior experience with alcohol, taking other drugs and medical conditions. | 1 |
| The first aider should be aware that symptoms of other medical conditions can mimic the symptoms of intoxication. | 1 |
| **Understanding alcohol intoxication** |  |
| The first aider should be aware that the body only metabolises approximately one standard drink of alcohol an hour. | 1 |
| The first aider should be aware that only time will reverse the effects of intoxication. | 1 |
| The first aider should be aware that drinking black coffee, sleeping, walking and cold showers will not reverse the effects of intoxication. | 1 |
| The first aider should be aware that intoxication may lead to a medical emergency. | 1 |
| The first aider should ask the intoxicated person if they have taken any medications or other drugs, in case the person’s condition deteriorates into a medical emergency. | 2 |
| The first aider should arrange for the intoxicated person to go to a hospital if they think the person is a risk to themselves. | 2 |
| The first aider should be aware that the person may be more intoxicated than they realise. | 2 |
| The first aider should be aware that when intoxicated the person may engage in a wide range of risky activities, such as having unprotected sex, vandalizing property or driving a car. | 1 |
| **What to do when the person is intoxicated** |  |
| The first aider should stay calm. | 1 |
| The first aider should stay with the intoxicated person or ensure they are not left alone. | 1 |
| The first aider should assess the situation for potential dangers and ensure that the intoxicated person, themselves and others are safe. | 1 |
| The first aider should monitor the intoxicated person and their environment to prevent tripping or falling. | 1 |
| The first aider should keep the intoxicated person away from machines and dangerous objects. | 1 |
| **Talking to the intoxicated person** |  |
| The first aider should talk with the intoxicated person in a respectful manner. | 1 |
| The first aider should talk with the intoxicated person using simple, clear language. | 1 |
| The first aider should be aware that the intoxicated person may overreact to negative words; therefore, the first aider should use positive words (such as ‘stay calm’) instead of negative words (such as ‘don’t fight’). | 1 |
| The first aider should not laugh at, make fun of, or provoke the intoxicated person. | 1 |
| The first aider should not attempt to engage the person in a serious conversation about their drinking behaviour while they are intoxicated. | 1 |
| **Getting the intoxicated person home** |  |
| The first aider should discourage the intoxicated person from driving a vehicle or riding a bike, for example, by telling them about the risks to both themselves and others. | 1 |
| The first aider should prevent the intoxicated person from driving a vehicle or riding a bike, but only if it is safe to do so. | 2 |
| If it is unsafe to prevent the person from driving, the first aider should call the police. | 3 |
| The first aider should organise a safe mode of transport for the intoxicated person to get home. | 1 |
| **What to do if the intoxicated person becomes aggressive** |  |
| The first aider should ensure their own safety at all times. | 1 |
| The first aider should de-escalate the situation as much as possible. | 1 |
| The first aider should remain as calm as possible. | 1 |
| The first aider should speak slowly and confidently. | 1 |
| The first aider should speak with a gentle, caring tone of voice. | 1 |
| The first aider should refrain from speaking to the intoxicated person in a hostile or threatening manner. | 1 |
| The first aider should avoid arguing with the intoxicated person. | 1 |
| The first aider should consider taking a break from the conversation to allow the intoxicated person a chance to calm down. | 2 |
| The first aider should watch the intoxicated person for signs of increasing aggression. | 1 |
| If violence has occurred, the first aider should seek the appropriate emergency assistance. | 1 |
| **Seeking medical help** |  |
| The first aider should not be afraid to seek medical help for the intoxicated person, if needed. | 1 |
| The first aider should seek medical help if required, even if there may be legal implications for the intoxicated person. | 1 |
| The first aider should be aware that it is beneficial for a friend or family member to accompany the intoxicated person to hospital because they may be able to provide relevant information. | 1 |
| **General principles for emergencies related to alcohol intoxication** |  |
| The first aider should be aware that alcohol consumption can mask pain from injuries. | 1 |
| The first aider should monitor the person’s airway, breathing and circulation. | 1 |
| If the intoxicated person stops breathing, the first aider should be aware they need expired air resuscitation (EAR). | 1 |
| If the intoxicated person has no pulse, the first aider should be aware that they need cardiopulmonary resuscitation (CPR). | 2 |
| The first aider should check for broken glass before rolling the person into the recovery position. | 1 |
| The first aider should keep the intoxicated person warm to prevent hypothermia (being aware that although the person may feel warm their body temperature may actually be decreasing). | 1 |
| **What to do if the intoxicated person vomits** |  |
| If the person has vomited, the first aider should clear the person’s airway if necessary. | 1 |
| If the person is vomiting and conscious, the first aider should keep the person sitting or put them in the recovery position. | 2 |
| If the person is continually vomiting, the first aider should call an ambulance. | 1 |
| If the person is vomiting and unconscious, the first aider should put them in the recovery position and stay with them until medical help arrives. If the person is left lying on their back they could suffocate on their vomit or their tongue could block their airway. | 1 |
| **What to do if the intoxicated person falls asleep** |  |
| If the intoxicated person is hard to wake, the first aider should put them in the recovery position. | 2 |
| If the intoxicated person cannot be woken, the first aider should call an ambulance. | 1 |
| **What to do if the person is dangerously intoxicated** |  |
| The first aider should know what alcohol poisoning means. | 1 |
| The first aider should be able to recognise the symptoms of alcohol poisoning. | 1 |
| The first aider should be aware that alcohol poisoning can lead to death. | 1 |
| The first aider should be aware that the amount of alcohol that causes alcohol poisoning is different for every person. | 1 |
| The first aider should take the intoxicated person to a hospital's emergency department if there is concern that their level of intoxication is dangerous. | 1 |
| The first aider should not wait for all the symptoms of alcohol poisoning to be present before getting medical help. | 1 |
| If alcohol poisoning is suspected, the first aider should ensure the intoxicated person is not left alone. | 1 |
| If alcohol poisoning is suspected, the first aider should not give the intoxicated person food as they may choke on it if they are not fully conscious. | 1 |
| If alcohol poisoning is suspected, the first aider should call an ambulance immediately. | 1 |
| The first aider should call an ambulance if the intoxicated person’s breathing is irregular, shallow or slow. | 1 |
| The first aider should call an ambulance if the intoxicated person’s pulse rate is irregular, weak or slow. | 1 |
| The first aider should call an ambulance if the intoxicated person’s skin is cold, clammy, pale or bluish in colour. | 1 |
| The first aider should call an ambulance if the intoxicated person falls unconscious. | 1 |
| **Other alcohol-related emergencies** |  |
| If the intoxicated person shows signs of a possible head injury (for example, they are vomiting and talking incoherently), the first aider should seek medical help. | 1 |
| If the intoxicated person expresses suicidal thoughts (e.g. states they wish to kill themselves) or demonstrates suicidal behaviour (e.g. tries to walk in front of cars), the first aider should call emergency services for assistance. | 2 |
| The first aider should seek medical help for the intoxicated person, if drink spiking is suspected. | 1 |
| **Alcohol withdrawal** |  |
| The first aider should know what alcohol withdrawal is. | 1 |
| The first aider should be able to recognise the symptoms of alcohol withdrawal. | 1 |
| The first aider should seek medical help if the person displays symptoms of severe alcohol withdrawal. | 1 |
| If the person stops drinking heavily, and becomes delirious and confused, the first aider should get medical help. | 1 |
| If the person stops drinking heavily, and begins hallucinating, the first aider should get medical help. | 1 |
| The first aider should seek medical help if the person has been drinking heavily for long periods and decides to stop suddenly. | 2 |
| The first aider should be aware that un-medicated alcohol withdrawal may lead to seizures. | 2 |
