# Supplementary material for: Helping someone with problem drinking: Mental health first aid guidelines - a Delphi expert consensus study
Source: BMC Psychiatry. 2009 Dec 7;9:79. doi: 10.1186/1471-244X-9-79 (PMC2799400; doi:10.1186/1471-244X-9-79)
Supplement: Additional file 2 — Helping someone with problem drinking: Mental Health First Aid guidelines. This file may be distributed freely, with the authorship and copyright details intact. Please do not alter the text or remove the authorship and copyright details. [file 1471-244X-9-79-S2.pdf]

MENTAL HEALTH FIRST AID

HELPING SOMEONE WITH PROBLEM DRINKING

MENTAL HEALTH FIRST AID GUIDELINES

What is problem drinking?

Problem drinking refers to drinking alcohol at levels that are associated with short-term and/or long-term harm. Such harms include:

- Family or social difficulties (e.g. relationship, work, financial problems)
- Non-fatal and fatal injuries (e.g. as a result of accidents, falls, violence, road trauma)
- Mental health problems (e.g. anxiety, depression)
- Physical health problems (e.g. ranging from nausea and headaches to stomach ulcers, liver or heart disease)

The person may have underlying reasons for drinking that need to be addressed. For example, they may be drinking to cope with problems in their life, such as relationship issues or untreated mental health problems. Mental health problems can be caused or exacerbated by drinking alcohol. Please see other guidelines in this series for further information on how to assist someone with different types of mental health problems.

Types of problem drinking

Someone may have a drinking problem if their drinking falls into one (or more) of the following categories:

1. **High-risk drinking** refers to drinking alcohol in amounts that exceed the low-risk recommendations in national drinking guidelines. It is important that you are familiar with the national guidelines for low-risk alcohol consumption in your country and how these guidelines define a *standard drink*. (A *standard drink* contains approximately 10g of alcohol; however the exact amount varies between countries.)

2. **Alcohol abuse** involves drinking which leads to one or more of the following problems occurring repeatedly in any 12 month period:

- Problems at work, school or home
- Legal problems
- Problems in social relationships
- Risk-taking activities while under the influence

3. **Alcohol dependence** involves three or more of the following in any 12 month period:

- Increased tolerance to the effects of alcohol (e.g. the person needs to drink more alcohol over time in order to feel its effects)
- Drinking more over longer periods than intended
- Problems in cutting down or controlling drinking
- A lot of time spent getting alcohol, drinking it, or recovering from its effects
- Missing important social, occupational or recreational activities because of drinking
- Withdrawal symptoms when attempting to stop or cut down
- Continuing to drink despite knowing about or suffering from its negative effects

Adapted from DSM-IV-TR, APA 2000

The MHFA Training & Research Program

Orygen Youth Health Research Centre
The University of Melbourne
AUSTRALIA

www.mhfa.com.au

MENTAL HEALTH FIRST AID

HELPING SOMEONE WITH PROBLEM DRINKING

MENTAL HEALTH FIRST AID GUIDELINES

Approaching someone about their drinking

If you are concerned about someone's drinking, talk to the person about it openly and honestly. Talk with them in a quiet, private environment at a time when there will be no interruptions, when both of you are sober and are in a calm frame of mind. Interact with the person in a supportive way, rather than threatening, confronting or lecturing them.

Try to understand the person's own perception of their drinking. Ask them about their drinking (for example, how much alcohol they tend to drink) and if they believe their drinking is a problem. Try to listen to the person non-judgmentally and avoid expressing moral judgments about their drinking. Do not accuse the person of being an alcoholic and avoid labelling them by using words such as "addict".

Consider the person's readiness to talk about their drinking by asking about areas of their life that it may be affecting, for example, their mood, work performance and relationships. Be aware that the person may not recognise or may deny that they have a drinking problem and that trying to force them to admit they have a problem may cause conflict.

Express your point of view by using "I" statements, for example, "I am concerned about how much you've been drinking lately". Identify and discuss the person's behaviour rather than criticise their character. For example, you could say "Your drinking seems to be getting in the way of your friendships" rather than saying "You're a pathetic drunk".

When discussing the person's drinking, bear in mind that they may recall events that occurred while they were drinking differently to how they actually happened. The person may not even recall some events that occurred while they were intoxicated.

Tell the person what you are willing and able to do to help. This may range from being a good listener to organising professional help. Do not expect a change in the person's thinking or behaviour right away. This conversation might be the first time they have thought about their drinking as a problem.

Encouraging the person to change their drinking

Although the person is the only one who can make the decision to change their drinking, it is both possible for the person to change their drinking habits and for you to assist them.

Changing drinking habits is not easy. You should know that:

- Willpower and self-resolve are not always enough to achieve change
- Advice alone may not help the person change their drinking
- The person may try to change or stop their drinking more than once before they are successful
- Many lifestyle changes are required to change drinking behaviours

Tell the person that only they can take responsibility for reducing their alcohol intake and that although changing drinking patterns is difficult, they should keep on trying.

If giving up alcohol altogether is not the person's goal, reducing the quantity of alcohol consumed is worthwhile. Ask the person if they would like some tips on low-risk drinking. If they do, tell them where they can access such information or suggest some tips from the box 'Practical tips for low-risk drinking'.

Reducing the risks associated with drinking

To reduce the risks associated with continuing to drink, encourage the person to find information on how to reduce the harms associated with problem drinking. Also, if appropriate, advise the person that alcohol may interact with illicit drugs or medications in an unpredictable way, which may lead to a medical emergency. If the person is pregnant or breast-feeding, advise them that not drinking at all is the safest way to avoid harming their baby.

Encouraging other supports

Encourage the person to reach out to friends and family who support their efforts to change their drinking. Let them know that not all family and friends will be supportive of their efforts to change and suggest they spend time with family and friends who do not drink. In addition, make the person aware of the range of non-professional supports available for problem drinking (e.g. self-help groups).

Practical tips for low-risk drinking

- Know how much alcohol is in a *standard drink*
- Know the number of *standard drinks* in each beverage (the number of *standard drinks* is often listed on the beverage's packaging)
- Keep count of the number of *standard drinks* consumed
- Do not let people top up your glass before it is finished, so as not to lose track of how much alcohol has been consumed
- Eat while drinking
- Drink plenty of water when drinking alcohol to prevent dehydration
- Drink beverages with lower alcohol content (e.g. low-alcohol beer instead of full strength beer)
- Switch to non-alcoholic drinks when starting to feel the effects of alcohol
- Avoid keeping up with friends drink for drink
- Avoid drinking competitions and drinking games
- Drink slowly, for example, by taking sips instead of gulps and putting the drink down between sips
- Only have one drink at a time
- Spend time in activities that don't involve drinking
- Drink alcohol as part of another activity instead of making it the main activity
- Identify situations where drinking is likely and avoid them if possible

HELPING SOMEONE WITH PROBLEM DRINKING

MENTAL HEALTH FIRST AID GUIDELINES

Managing social pressure to drink

There is often social pressure to get drunk when drinking. Advise the person to be assertive when they feel pressured to drink more than they want or intend to. Tell the person that they have the right to refuse alcohol. Suggest different ways the person can say no, such as saying "no thanks" without explanation, "I don't want to", "I don't feel well" or "I am taking medication". Encourage the person to practise different ways of saying "no". Suggest to the person that the people who care about them will accept their decision not to drink or to reduce the amount that they drink.

What if the person is unwilling to change their drinking?

If the person is unwilling to change their drinking, you can speak with a health professional to determine how best to approach the person about your concerns, or you could consult with others who have dealt with problem drinking about effective ways to help. Another strategy is to discuss with the person the link between their drinking and the negative consequences they are experiencing. Do not feel guilty or responsible if the person is unwilling to change their drinking.

If the person is unwilling to change their drinking, do not:

- Join in drinking with the person
- Try to control the person by bribing, nagging, threatening or crying
- Make excuses for the person or cover up their drinking or related behaviour
- Take on the person's responsibilities, except if not doing so would cause harm (e.g. to their own or others' lives)

Professional help

When to seek professional help?

The person may need to seek professional help for their drinking if they:

- Acknowledge they think a lot about alcohol and when they'll next get a chance to drink
- Become anxious when they cannot get access to alcohol
- Need alcohol to help deal with certain situations
- Get into arguments or have accidents because of alcohol
- Have difficulty performing day-to-day tasks as a result of their drinking
- Are in debt because of the amount of money they spend on alcohol

Offer to support the person in getting professional help. If they are willing to seek professional help, give them information about local options and encourage them to make an appointment.

What if the person does not want professional help?

When professional help is first suggested, the person may find it difficult to accept they need help or they may not want assistance. If the person won't seek help because they don't want to stop drinking completely, explain to them that there are several approaches available for treating drinking problems and that the treatment goal may be to reduce alcohol consumption rather than to quit altogether. Reassure the person that professional help is confidential.

If the person is still unwilling to seek professional help, you should set limits around what behaviour you are willing and not willing to accept from them. It is important to continue to suggest professional help if they are putting themselves or others at risk of harm.

Be prepared to talk to the person about seeking professional help in the future. Be compassionate and patient while waiting for them to accept they need help. Seeking professional help is ultimately the person's decision. They cannot be forced, except under certain circumstances, for example, if a violent incident results in the police being called or following a medical emergency.

First aid for alcohol intoxication, poisoning or withdrawal

Alcohol intoxication

Alcohol intoxication refers to significantly elevated levels of alcohol in the blood that substantially impairs the person's thinking and behaviour. While intoxicated, the person may engage in a wide range of risky activities (such as unprotected sex, fighting or driving a car).

Signs of alcohol intoxication include:

- Loss of coordination
- Slurred speech
- Staggering or falling over
- Loud, argumentative or aggressive behaviour
- Vomiting
- Drowsiness or sleepiness

The signs of alcohol intoxication can vary from person to person and are influenced by a range of factors, such as prior experience with alcohol, taking illicit drugs or medications, and physical and mental health conditions. The symptoms of some medical conditions can mimic the signs of alcohol intoxication.

Alcohol poisoning and alcohol withdrawal

Alcohol poisoning refers to a toxic level of alcohol in the blood that can lead to death. The amount of alcohol that causes poisoning is different for every person.

Alcohol withdrawal refers to a range of symptoms that may occur when a person who has been drinking heavily on a regular basis stops drinking or drinks substantially less than usual. It is not simply a hangover.

What to do if the person is intoxicated

Stay calm and communicate appropriately

Talk with the person in a respectful manner, using simple, clear language. Do not laugh at, make fun of, or provoke the person. Do not attempt to engage the person in a serious conversation about their drinking while they are intoxicated. If the person becomes aggressive, there are some additional things you should consider (see the box, 'What to do if the person is aggressive').

Monitor for danger

Assess the situation for potential danger and ensure that the person, yourself and others are safe. Be aware that the person may be more intoxicated than they realise. While intoxicated, the person is at greater risk of physical or sexual assault. Monitor the person and their environment to prevent tripping or falling. Ask the person if they have taken any medications or other drugs, in case their condition deteriorates into a medical emergency. Also be aware that alcohol consumption can mask pain from injuries. Watch the person for signs of increasing aggression.

Keep the person safe

Stay with the person or ensure they are not left alone. Keep them away from machines and dangerous objects. If the person attempts to drive a vehicle or ride a bike, discourage them (for example, by telling them about the risks to both themselves and others). Only prevent the person from driving if it is safe to do so. If it is unsafe, call the police.

If the person expresses suicidal thoughts or demonstrates suicidal behaviour, call emergency services for assistance. For more information, please see the other guidelines in this series: *Suicidal thoughts and behaviours: first aid guidelines.*

If you think the person is a risk to themselves, arrange for them to go to a hospital; otherwise organise a safe mode of transport to get the person home.

Can I help the person sober up?

Only time will sober the person up. The body breaks down approximately one *standard drink* of alcohol per hour. Drinking black coffee, sleeping, walking and taking cold showers will not speed up this process.

What to do if the person is aggressive

If the person becomes aggressive, assess the risks to yourself, the person and others. Ensure your own safety at all times so that you can continue to be an effective helper. If you feel unsafe, seek help from others. Do not stay with the person if your safety is at risk. Remain as calm as possible and try to de-escalate the situation with the following techniques:

- Talk in a calm, non-confrontational manner
- Speak slowly and confidently with a gentle, caring tone of voice
- Try not to provoke the person; refrain from speaking in a hostile or threatening manner and avoid arguing with them
- Use positive words (such as “stay calm”) instead of negative words (such as “don’t fight”) which may cause the person to overreact
- Consider taking a break from the conversation to allow the person a chance to calm down
- Try to provide the person with a quiet environment away from noise and other distractions
- If inside, try to keep the exits clear so that the person does not feel penned in and you and others can get away easily if needed

If violence has occurred, seek appropriate emergency assistance.

Medical emergency

It is important to know that alcohol intoxication, poisoning and withdrawal may lead to a medical emergency.

Signs of an alcohol-related medical emergency

- The person is continually vomiting
- The person is unconscious i.e. falls asleep and cannot be woken
- Alcohol poisoning is suspected i.e. the person shows any of the following signs or symptoms:
  - irregular, shallow or slow breathing
  - irregular, weak or slow pulse rate
  - cold, clammy, pale or bluish coloured skin
- Drink spiking is suspected (e.g. the person shows a rapid increase in alcohol intoxication)
- The person has a suspected head injury
- The person shows signs of severe alcohol withdrawal, which include:
  - developing a fever
  - being delirious or confused
  - developing convulsions or seizures
  - experiencing hallucinations

HELPING SOMEONE WITH PROBLEM DRINKING

MENTAL HEALTH FIRST AID GUIDELINES

MENTAL HEALTH FIRST AID

What to do in a medical emergency

In a medical emergency, call an ambulance or take the person to a hospital. Do not be afraid to seek medical help for the person, even if there may be legal implications for them.

If an ambulance is called or medical help is sought, ensure that the person:

- Is not left alone
- Is not given food or drink as they may choke on it if they are not fully conscious
- Is kept warm to prevent hypothermia (although the person may feel warm, their body temperature may actually be decreasing)
- Has their airway, breathing and circulation monitored
- Is put in the recovery position if they are hard to wake (see the box 'The recovery position')

It is beneficial for a friend or family member to accompany the person to hospital as they may be able to provide relevant information.

Other first aid principles to keep in mind:

- If the person is vomiting and conscious keep them sitting. Alternatively, put them in the recovery position
- If the person stops breathing, they will need expired air resuscitation (EAR)
- If the person has no pulse, they will need cardiopulmonary resuscitation (CPR)

The recovery position

Any unconscious person needs immediate medical attention and their airway kept open. If they are left lying on their back they could suffocate on their vomit or their tongue could block their airway. Check for broken glass and other sharp objects on the ground before rolling them into the recovery position. Putting the person in the recovery position will help to keep the airway open. If necessary, clear the person's airway after they have vomited.

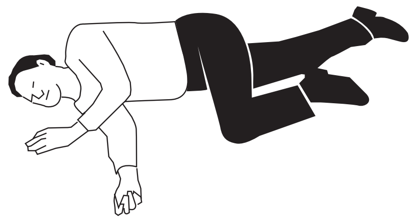

Purpose of these Guidelines

These guidelines are designed to help members of the public to provide first aid to someone who may have a drinking problem. The role of the first aider is to assist the person until appropriate professional help is received, if needed.

Development of these Guidelines

The following guidelines are based on the expert opinions of a panel of mental health consumers, carers and clinicians from Australia, Canada, Ireland, New Zealand, the USA and the UK about how to help someone who is developing or experiencing a drinking problem. Details of the methodology can be found in: Kingston, A.H., Jorm, A.F., Kitchener, B.A., Hides, L., Kelly, C.M., Morgan, A.M., Hart, L.M., & Lubman, D.I. (2009) *Helping someone with problem drinking: mental health first aid guidelines – a Delphi expert consensus.*

How to use these Guidelines

These guidelines are a general set of recommendations about how you can help someone who may have a drinking problem. Each individual is unique and it is important to tailor your support to that person's needs. These recommendations therefore may not be appropriate for every person with problem drinking. Problems with alcohol may occur at the same time as problems with other drugs or mental health issues. Also, the guidelines are designed to be suitable for providing first aid in developed English-speaking countries. They may not be suitable for other cultural groups or for countries with different health systems.

Problem drinking may occur in the context of other drug use or mental health problems, and heavy alcohol use is strongly associated with suicidal behaviour. If you are concerned that the person may also have a problem with other drugs (such as cannabis, ecstasy, amphetamines, cocaine and/or heroin) please see *Helping someone with problem drug use: mental health first aid guidelines.* If you think the person may be depressed or suicidal, please see *Depression: first aid guidelines or Suicidal thoughts or behaviours: first aid guidelines.*

Although these guidelines are copyright, they can be freely reproduced for non-profit purposes provided the source is acknowledged. Please cite these guidelines as follows:

Mental Health First Aid Training and Research Program. *Helping someone with problem drinking: mental health first aid guidelines.* Melbourne: Orygen Youth Health Research Centre, University of Melbourne. 2009.

Enquiries should be sent to:

Professor Tony Jorm, Orygen Youth Health Research Centre
Locked Bag 10, Parkville VIC 3052 Australia
email: ajorm@unimelb.edu.au

All MHFA guidelines can be downloaded from **www.mhfa.com.au**

8252_09_ORYGENKJ
